# The Role of the Core Non-Homologous End Joining Factors in Carcinogenesis and Cancer

**DOI:** 10.3390/cancers9070081

**Published:** 2017-07-06

**Authors:** Brock J. Sishc, Anthony J. Davis

**Affiliations:** Department of Radiation Oncology, University of Texas Southwestern Medical Center, Dallas, TX 75390, USA; brock.sishc@utsouthwestern.edu

**Keywords:** NHEJ, cancer, genomic instability, carcinogenesis, cancer therapy, DSB repair

## Abstract

DNA double-strand breaks (DSBs) are deleterious DNA lesions that if left unrepaired or are misrepaired, potentially result in chromosomal aberrations, known drivers of carcinogenesis. Pathways that direct the repair of DSBs are traditionally believed to be guardians of the genome as they protect cells from genomic instability. The prominent DSB repair pathway in human cells is the non-homologous end joining (NHEJ) pathway, which mediates template-independent re-ligation of the broken DNA molecule and is active in all phases of the cell cycle. Its role as a guardian of the genome is supported by the fact that defects in NHEJ lead to increased sensitivity to agents that induce DSBs and an increased frequency of chromosomal aberrations. Conversely, evidence from tumors and tumor cell lines has emerged that NHEJ also promotes chromosomal aberrations and genomic instability, particularly in cells that have a defect in one of the other DSB repair pathways. Collectively, the data present a conundrum: how can a single pathway both suppress and promote carcinogenesis? In this review, we will examine NHEJ’s role as both a guardian and a disruptor of the genome and explain how underlying genetic context not only dictates whether NHEJ promotes or suppresses carcinogenesis, but also how it alters the response of tumors to conventional therapeutics.

## 1. Introduction

A myriad of elegant mechanisms have evolved that repair the vast number of DNA lesions an organism encounters each day. These repair pathways are critical because faithful propagation of genetic material and transmission to daughter cells is required for life; therefore, DNA repair mechanisms are described as guardians of the human genome [1]. Arguably, the most important DNA repair mechanisms are those that repair DNA double-strand breaks (DSBs). DSBs are the most toxic DNA lesion, because if they are unrepaired, they can drive apoptosis or senescence, and if they are misrepaired they can lead to the generation of chromosomal aberrations, resulting in genomic instability, which can lead to tumorigenesis [2]. The pathways that repair DSBs in human cells are homologous recombination (HR), non-homologous end joining (NHEJ), and an alternative end joining pathway (Alt-EJ) [3]. HR directs repair by using a homologous DNA sequence as a template to guide error-free restoration of the DNA molecule. HR is primarily active in mid-S phase to early G2 phase of the cell cycle, because an accessible homologous template to mediate repair is readily available via a sister chromatid in these cell cycle phases. NHEJ directly re-ligates the two broken DNA strands using a template-independent mechanism, and since it does not require a homologous template NHEJ is not restricted to a particular cell cycle phase. Alt-EJ is primarily a back-up pathway for both HR and NHEJ and it typically utilizes microhomologies distant from the DSB site to drive repair, generally leading to excessive end resection and mutation events.

In this review, we will focus on the role of NHEJ in genomic maintenance and cancer incidence. NHEJ is the predominant repair pathway in human cells and has traditionally been regarded as a guardian of the genome, preventing genomic instability through the repair of DSBs. This viewpoint is supported by the fact that loss of the core NHEJ factors, Ku70/80 (Ku), DNA-dependent protein kinase catalytic subunit (DNA-PKcs), DNA Ligase IV (LIG4), X-ray repair cross-complementing protein 4 (XRCC4), and XRCC4-like factor (XLF, also known as Cernunnos), leads to genomic instability, indicated by an increased frequency in chromosomal translocations and aberrations, including small deletions or insertions. Furthermore, NHEJ defects result in increased sensitivity of cells to genotoxic agents including ionizing radiation (IR) and chemotherapeutics. While the role of NHEJ in guarding the genome and protecting cells from agents that induce DSBs is well established, evidence has emerged that deregulated NHEJ may also promote carcinogenesis by increasing genomic instability due to inappropriate repair. These dueling phenotypes for NHEJ-mediated DSB repair present a paradox: how can a single pathway be both a guardian, thereby preventing genomic instability and thus tumorigenesis, and a driver, thereby promoting genomic instability, and by proxy, carcinogenesis? In this review, we will explore and seek to reconcile this paradox by presenting a comprehensive overview of the literature examining the role that the core NHEJ factors, Ku70 (*XRCC6*), Ku80 (*XRCC5*), DNA-PKcs (*PRKDC* or *XRCC7*), LIG4 (*LIG4*), XRCC4 (*XRCC4*), and XLF (*NHEJ1*), play in protecting the genome and how dysregulation of these factors influences carcinogenesis, cancer progression and aggressiveness, and patient survival. Furthermore, we will examine targeting NHEJ as a therapeutic cancer treatment strategy.

## 2. NHEJ General Mechanism

**Figure 1:**
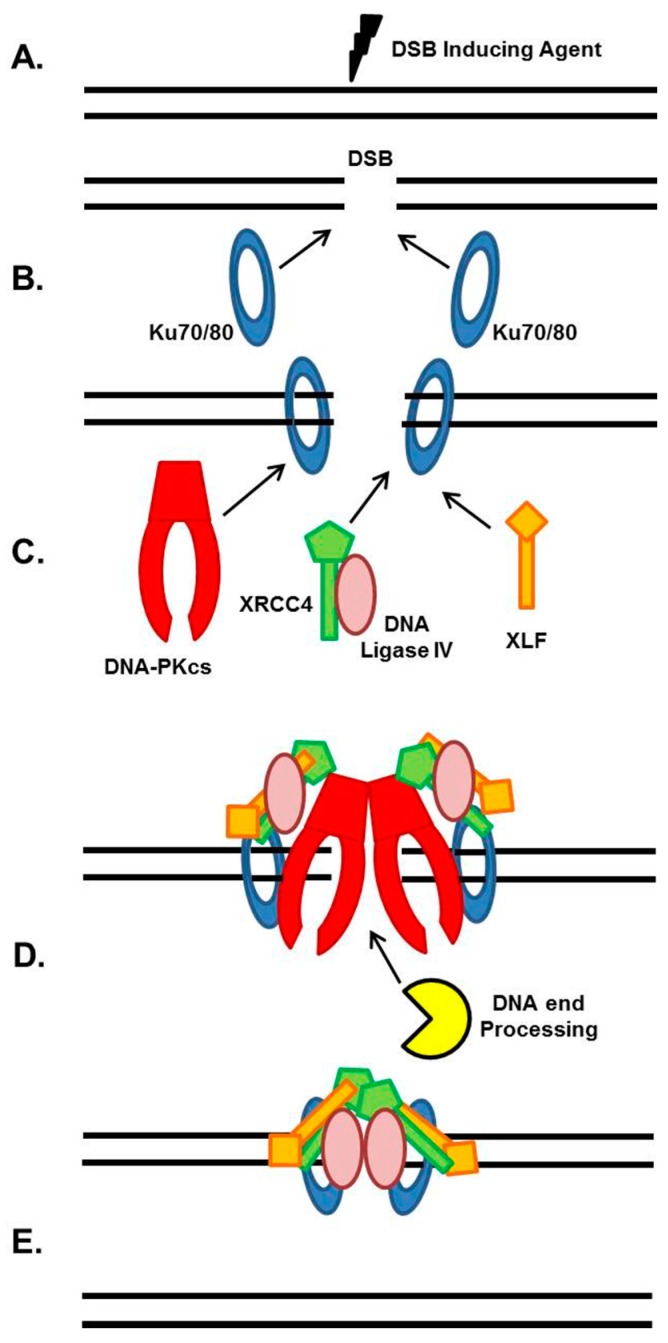
General NHEJ Mechanism. (**A**) and (**B**). A DNA double strand break (DSB) is induced and is quickly bound by the Ku heterodimer; (**C**). Ku70/80 serves as a scaffold to recruit the NHEJ machinery to the DSB; (**D**). If the DSB ends cannot be ligated, they will be processed by specific DNA end processing factors; (**E**). The DSB is ligated by DNA Ligase IV and NHEJ is complete.

NHEJ is the major pathway responsible for the repair of ionizing radiation (IR)-induced DSBs and DSBs intentionally generated for V(D)J and class switch recombination during T- and B-cell lymphocyte maturation [4,5,6]. The strengths of the NHEJ pathway are that it directs re-ligation of the broken DNA molecule in a template-independent manner, and is active in all phases of the cell cycle. Although NHEJ is traditionally characterized as the repair mechanism that simply rejoins the broken DNA ends regardless of the genetic sequence at the break, it is actually a flexible and dynamic process that can respond to variable types of DSBs [5,7,8]. The intricate and detailed mechanism of NHEJ has been covered in previous reviews, so we will only briefly explain the general mechanism, which is outlined in Figure 1 [5,6,7,8]. NHEJ initiates when the ring-shaped Ku heterodimer, composed of the Ku70 and Ku80 proteins, recognizes and rapidly binds to the DSB in a sequence-independent manner (Figure 1A,B) [9]. Once bound to the DSB ends, Ku then performs its primary function as a scaffold to recruit the NHEJ machinery to the damage site (Figure 1C). In particular, Ku70/80 directly recruits DNA-PKcs to the DNA ends to form the active DNA-PK complex, resulting in activation of DNA-PKcs kinase activity [5]. DNA-PKcs is a nuclear serine/threonine kinase that belongs to the phosphatidylinositol-3-kinase (PI3K)-related kinase (PIKK) family, which also includes the DNA damage responsive kinases ataxia telangiectasia mutated (ATM) and ataxia telangiectasia and Rad3 (ATR). DNA-PKcs kinase activity is required for NHEJ, but it is not completely understood what role DNA-PKcs activity plays in the process. If the ends of the DSB are not compatible for ligation, different end processing enzymes are required, including those that resect DNA ends, fill in gaps, or remove blocking end groups, to process the DNA ends to allow ligation (Figure 1D). The enzymes responsible for processing DNA ends for the NHEJ pathway include Artemis, Polynucleotide Kinase 3′-Phosphatase (PNKP), Aprataxin and PNK-like factor (APLF), Polymerases μ and λ, Werner (WRN), and Ku [5]. The terminal step in NHEJ is ligation of the broken DNA ends by DNA Ligase IV (Figure 1E). LIG4 is an ATP-dependent DNA ligase that uses ATP to adenylate itself and then transfers the AMP group to the 5′ phosphate of one end of the DSB [10]. There is a nucleophilic attack by the 3′ hydroxyl group of the second DSB end, and release of AMP yields the ligation product. XRCC4 stabilizes LIG4 protein in cells and may also enhance end joining by promoting DNA end bridging via its ability to form long filaments with XLF [10,11,12]. XLF stimulates LIG4-mediated ligation via promoting re-adenylation of LIG4 [5,10]. LIG4 is a flexible enzyme as it can ligate incompatible DNA ends and across gaps and XLF stimulates the activity of LIG4 towards mismatched and non-cohesive DNA ends [5,13].

## 3. NHEJ’s General Role in Genome Maintenance and Instability

A broad range of genomic rearrangements, including deletions, amplifications, insertions, inversions, and reciprocal and nonreciprocal translocations events, are believed to be among the drivers of carcinogenesis [14,15]. NHEJ protects the genome from chromosomal aberrations, which is supported by the observations that human and rodent cells lacking NHEJ factors are highly sensitive to agents that cause DSBs and have elevated levels of spontaneous and damage-induced genomic rearrangements [16,17,18]. Although NHEJ has traditionally been considered an error-prone process, in humans it is likely that NHEJ is not intrinsically inaccurate and that the quality of the end-joining is dictated by the structure of the DSB ends and not due to the inadequacy of the NHEJ machinery [19]. For example, NHEJ can mediate precise end-joining of DSBs, such as those with blunt ends or perfectly cohesive ends. Furthermore, LIG4 promotes precise end-joining by limiting end-processing and errors via its ability to ligate across small (1–2 bp) gaps or damaged termini [20]. NHEJ’s role in supporting genome maintenance is also supported by the fact that unrepaired DSBs (DNA fragments) and dicentric chromosomes increase as DNA-PK activity decreases [21]. Furthermore, decreased NHEJ-mediated DNA end joining capacity is associated with increased cancer incidence, likely a result of increased activity of the highly error-prone Alt-EJ pathway [22,23,24]. Finally, DSB repair by NHEJ is lower and the aberrant Alt-EJ pathway is higher as mice age, suggesting that decreased NHEJ may be the mechanism responsible for age-related genomic instability and increased cancer incidence [25].

**Figure 2:**
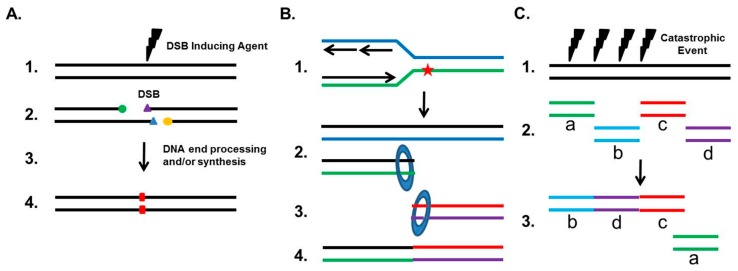
NHEJ-mediated chromosomal aberrations. (**A**). If the two junctions of the DSB (1.) are incompatible for ligation (2.), they will be processed (3.), which can result in small deletions, insertions via the fill-in polymerases, or indels (designated as a red rectangle) (4.); (**B**). Homologous recombination typically directs the repair of one-ended DSBs, which arise when a replication fork collapses or when the replication fork hits a DNA lesion (designated as a red star) (1.). However, if NHEJ attempts to repair one-ended DSBs (2.), it will do so by using a distal DSB to mediate repair (3.), resulting in a translocation (4.); (**C**). A single catastrophic event, termed chromothripsis (1.), can produce multiple DSBs of a chromosome (2.), which are then randomly rejoined by NHEJ in a chaotic genomic structure (3.).

Although NHEJ plays a role as a guardian of the human genome, it can also drive genomic rearrangements (outlined in Figure 2). If the two junctions of the DSB are incompatible for ligation and must be processed, NHEJ can result in small deletions (typically 1–4 bp), insertions via the fill-in polymerases, or indels (Figure 2A). A well-studied genomic rearrangement that is associated with the etiology of carcinogenesis is the chromosomal translocation. Chromosomal translocations typically result when two independent DSBs occur on different chromosomes, thereby generating four total DNA ends. If the correct DNA ends are rejoined, then no translocation occurs, but if ends from different chromosomes are joined, then a translocation develops. Studies, using nucleases to introduce site-specific DSBs at endogenous loci, translocation reporter assays, and DSBs induced by radiation, have all found that NHEJ is responsible for the generation of translocations in human cells [17,26,27,28]. This is in contrast to data from mouse cells, which show that translocations are suppressed by NHEJ and arise from Alt-EJ [29]. To date, 300+ known chromosomal translocations have been identified in hematological disorders and malignant solid tumors, and a number of studies have found that the NHEJ pathway is likely responsible for these translocations in multiple human cancers. For example, androgens and genotoxic stress synergistically induce cancer-specific translocations in prostate cancer cells, with the fusions requiring the NHEJ pathway [30]. Furthermore, NHEJ is responsible for the translocations found in human lymphoid cells and renal cell carcinoma (RCC), as the junctions of the translocations have the features of NHEJ-mediated repair, including small deletions near the DSB sites and template-independent nucleotide insertion by either DNA polymerase μ or Pol λ [31,32].

It has also become increasingly apparent that inappropriate NHEJ drives genomic rearrangements and instability. One-ended DSBs, which arise when a replication fork collapses or when the replication machinery encounters a DNA lesion such as a single-strand break or a DNA cross-link, are preferentially repaired by HR in S phase. Any attempt by NHEJ to repair these one-ended DSBs will result in mis-joining, because NHEJ has to utilize another DSB, typically from a distal site, resulting in a translocation (Figure 2B). This commonly occurs when the cell has a defect in HR, and aberrant NHEJ results in chromatid fusions between heterologous chromosomes. Chromosomal abnormalities and repair defects of the cancer predisposition syndrome Fanconi Anemia (FA), which is required for the repair of DNA cross-links, result from the inappropriate action of NHEJ at damaged/stalled replication forks in S phase. Cytological studies in FA cells show that the vast majority of radial chromosomes generated in response to DNA cross-links arise as a result of translocations between non-homologous chromosomes, which is a signature of repair by NHEJ [33,34]. Furthermore, eliminating NHEJ substantially rescues the intrastrand crosslink repair deficit associated with FA-deficient cells. It is believed that the FA pathway blocks NHEJ from engaging one-sided DSBs at replications forks by preventing the inappropriate engagement of the DNA-PK complex with damaged replication forks to divert DSB repair toward the HR pathway. Loss of the FA pathway in head and neck squamous cell carcinoma (HNSCC) cells promotes cellular invasion and this response requires DNA-PKcs activity [35]. Finally, recent evidence has emerged that a single catastrophic event, termed chromothripsis, that results in multiple DSBs of a chromosome are randomly rejoined by NHEJ in a chaotic genomic structure (Figure 2C) [36]. Recent estimates are that up to 5% of all tumors show evidence of chromothripsis, and it has been found to be elevated in specific tumor types, including bone cancers and late-stage neuroblastomas [37]. It has been postulated that chromothripsis drives carcinogenesis via the loss of a tumor suppressor gene, oncogenic gene fusions, or via oncogene amplification through the formation of double-minute chromosomes during the repair of the shattered chromosome [37,38]. Interestingly, DNA-PKcs-dependent NHEJ plays a role in the formation of double minutes in colon cancer cells, which suggests that NHEJ may be responsible for the complete oncogenic potential of chromothripsis [39].

## 4. Core NHEJ Factors in Carcinogenesis and Cancer

In this section, we will examine the significant amount of literature exploring the role of differential regulation and single nucleotide polymorphisms (SNPs) in the core NHEJ genes that correlate with cancer incidence, tumor aggressiveness, and responses to conventional radiation and chemotherapies.

### 4.1. Rodent Models and Human Patients with Defects in the Core NHEJ Factors

A significant amount of genetic evidence from rodent models shows that loss or mutation of the core NHEJ factors has significant physiologic consequences, including increased genomic instability and carcinogenesis. Ku-deficient cells and mice display inaccurate end-joining, dramatic radiosensitivity, and genomic instability, including chromosomal breakage, translocations, and aneuploidy [40,41,42,43,44,45]. Additionally, both Ku70^−/−^ and Ku80^−/−^ mice display decreased size, defects in V(D)J recombination and B- and T-lymphocyte maturation, and premature aging [40,42,46,47,48,49]. Ku70^−/−^ mouse fibroblasts have an increased rate of sister chromatid exchange (SCE) and a high frequency of spontaneous neoplastic transformation [47]. Ku70^−/−^ mice develop thymic and disseminated T-cell lymphomas [46,47]. Ku80^−/−^ combined with p53^−/−^ results in early incidence of pro-B-cell lymphoma that resembles Burkitt’s lymphoma [43]. Haplo-insufficiency of Ku80 in PARP^−/−^ mice promotes the development of hepatocellular adenoma and hepatocellular carcinoma (HCC) [50]. Cytogenetic analysis revealed that Ku80 heterozygosity elevated chromosomal instability in PARP^−/−^ cells and that liver tumors isolated from these mice harbored a high degree of chromosomal aberrations, including fragments, end-to-end fusions, and recurrent nonreciprocal translocations; features reminiscent of human HCC [50]. DNA-PKcs-deficient rodent cells are extremely radiosensitive and are defective in the repair of DSBs [5,6]. DNA–PKcs is mutated in mice with severe combined immune-deficiency (SCID), and these mice and the cells derived from them are radiosensitive, have defective V(D)J recombination, shortened telomeres, and accelerated aging [51,52,53,54,55]. DNA-PKcs^−/−^ mice present with lymphomas and preneoplastic lesions in the intestinal mucosa and production of aberrant crypt foci, suggesting that DNA-PKcs protects against tumorigenesis [53,56]. Furthermore, rapid onset of lymphomas and leukemias was observed in mice with the DNA-PKcs SCID mutant background in the absence of p53 [57]. BALB/c mice carry a defect in DNA DSB rejoining that is quantitatively different from that of SCID mice, and it was found that two mutations in the mouse *PRKDC* gene results in reduced DNA-PKcs expression and activity [58,59]. There is an elevated breast cancer risk in irradiated BALB/c mice, suggesting that DNA-PKcs protects mice from tumorigenesis [59]. Blocking phosphorylation of DNA-PKcs at the threonine 2609 cluster in mice results in congenital bone marrow failure, and rescue of these mice with bone marrow transplants results in spontaneous tumor development [60,61]. LIG4 null mice (LIG4^−/−^) are embryonic lethal with the mice showing widespread neural apoptosis [62]. p53 deficiency (p53^−/−^) rescues this embryonic lethality, and LIG4^−/−^p53^−/−^ mice develop medulloblastoma and pro-B lymphomas [63,64]. Using the tumor-prone ink4a/arf^−/−^ mouse strain, it was found that a loss of a single copy of *LIG4* promotes development of soft tissue sarcomas that possess clonal amplifications, deletions, and translocations [65]. Absence of XRCC4 in rodent cell lines leads to radiation sensitivity and defects in DSB repair and V(D)J recombination [66]. Similar to LIG4^−/−^ mice, XRCC4 null mice (XRCC4^−/−^) present with increased neuronal apoptosis, embryonic lethality, and impaired cellular proliferation, with p53 deficiency rescuing these phenotypes [67]. XRCC4^−/−^ mouse embryonic fibroblasts (MEFs) exhibit marked genomic instability, including chromosomal translocations, and XRCC4^−/−^p53^−/−^ mice succumb to pro-B-cell lymphomas, which have increased chromosomal translocations [67]. Conditional inactivation of *XRCC4* in nestin-expressing neuronal progenitor cells in a p53^−/−^ background results in early onset of neuronally differentiated medulloblastomas, and these medulloblastomas show recurrent clonal translocations [68]. XLF-deficient MEFs are radiosensitive and are severely impaired in their ability to mediate V(D)J recombination, but. mature lymphocyte numbers in XLF^−/−^ mice are only modestly decreased and pro-B lines show V(D)J recombination at nearly wild-type levels [69]. XLF^−/−^p53^−/−^ mice develop medulloblastomas but are not prone to the pro-B lymphomas that occur in Lig4^−/−^p53^−/−^ and XRCC4^−/−^p53^−/−^ mice [69]. In mouse models, the data clearly shows that the core NHEJ factors promote genomic stability and protect against carcinogenesis.

Conversely, only a limited number of human patients have been identified that have a loss or a verified disease-causing mutation in a core NHEJ factor. No human patient has been identified with a verified disease-causing mutation or loss of Ku, but knock-out of Ku70 or Ku80 in human cells results in cell death, which is believed to be due to rapid loss of telomere length [70,71]. A few human patients have been identified with mutations in DNA-PKcs. The initial patient presented with radiosensitive T^−^B^−^ severe SCID, and cells isolated from the patient show a defect in overall end joining [72]. A second patient with a *PRKDC* mutation presenting with SCID and defective DSB repair also has profound neurological abnormalities [72,73]. Recently, a patient with mutations in the *PRKDC* gene was discovered who had immunodeficiency, granuloma, and autoimmune regulator-dependent autoimmunity [74]. Finally, a patient with xeroderma pigmentosum (XP) was also found to be radiosensitive due to a splice variant of DNA-PKcs in which exon 31 was deleted [75]. A glioma cell line, M059J, was identified that is deficient for DNA-PKcs, and this cell line exhibits a radiosensitive phenotype and is defective in repair of DSBs [76,77]. However, it should be noted that this is the only human cancer cell line found with a complete loss of DNA-PKcs. Mutations in *Lig4* are linked to Ligase IV syndrome, a disorder associated with microcephaly, severe immunodeficiency, cell radiosensitivity, and chromosome instability [78,79]. Mutations in *LIG4* are also associated with DNA repair defects in a case of Dubowitz Syndrome [80]. The 180BR cell line derived from a radiosensitive leukemia patient is characterized by the R278H mutation residing in the catalytic center of LIG4 that leads to impaired activity of the mutated enzyme [81]. A patient with microcephaly and progressive ataxia but a normal immune response has been identified with mutations in the *XRCC4* gene [82]. The patient’s cells from this XRCC4 defective patient are radiosensitive and display a severe DSB repair defect. XLF was initially identified in five patients with growth retardation, microcephaly, and immunodeficiency characterized by T+B lymphocytopenia [83]. Mutations affecting the *NHEJ1* gene were identified in all five patients, and fibroblasts obtained from the patients showed an increased cellular sensitivity to ionizing radiation, defective V(D)J recombination, and an impaired DNA-end ligation process [83]. A predisposition to malignancies has been found in approximately 25% of LIG4 syndrome patients, suggesting that NHEJ likely does play a role in protecting against genomic instability and carcinogenesis in humans [84].

### 4.2. Differential Expression of the Core NHEJ Factors and Carcinogenesis

One potential way to drive chromosomal instability and thus promote carcinogenesis would be for a cell to downregulate NHEJ. However, downregulation of the core NHEJ factors has been found in only a small number of human cancers. Ku70 and Ku80 expression was reported in two studies to be significantly reduced in colon cancer, with this decrease in Ku associated with chromosomal instability [85,86]. Ku70 expression was also reported to be lower in endometrioid endometrial cancer and Waldentrom’s acroglobulinemia [87,88]. One study found that when comparing healthy and breast tumor tissue proteomes, Ku70, Ku80, and DNA-PKcs expression was significantly downregulated in the tumor tissues [89]. Peripheral blood lymphocytes (PBL) from patients with uterine, cervix, or breast cancer had significantly decreased DNA-PK activity and higher rates of chromosome aberrations, relative to normal volunteers [90]. Finally, reduced DNA-PKcs-mediated repair activity was reported to be associated with an increased risk for lung cancer [91].

Another possible mechanism for promoting chromosomal instability would be to have NHEJ that is overly active via overexpression of the core NHEJ factors. A panel of myeloid leukemias was found to have large deletions, which were associated with increased NHEJ activity [23]. The authors speculated that Ku drives an overactive NHEJ system and that this aberrant Ku activity is a candidate mechanism for inducing chromosomal instability and carcinogenesis [23]. Increased Ku protein levels, DNA-binding activity, and substantial activation of the NHEJ pathway were also found in colorectal tumors [92]. Ku70 protein levels in precancerous lesions and gastric cancer tissues were significantly higher than in normal gastric mucosa tissues [93]. Furthermore, *Ku70* gene expression in poorly-differentiated colorectal tumors was reported to be significantly higher than in well- and moderately-differentiated tumors [94]. A number of studies have found a correlation between increased expression of DNA-PKcs and carcinogenesis. Expression of *PRKDC* mRNA and protein was notably higher in prostate cancer tissues than in normal tissues and was proposed to promote prostate tumorigenesis [95]. *PRKDC* was also found to be up-regulated in colorectal cancerous tissues compared to normal tissues [96,97]. At the mRNA level, DNA-PKcs expression was significantly higher in non-small cell lung carcinoma (NSCLC) tumor tissues than in the adjacent normal tissues [98]. Aberrant expression of DNA-PKcs conveys oncogenic properties in HCC with upregulation of DNA-PKcs significantly elevated in tumors compared to normal tissue [99], and a second study classified DNA-PKcs as a candidate driver in hepatocarcinogensis, with amplification of the *PRKDC* locus and increased mRNA expression found in HCC [100]. DNA-PKcs activity was higher in esophageal tumor tissues than in the adjacent normal mucosae, however DNA-PK protein expression displayed intratumoral heterogeneity [101]. Finally, DNA-PKcs was found to be overexpressed in gastric cancer tissues compared to wild-type tissues [102].

There are limited data in regards to the expression of LIG4, XRCC4, and XLF in potentially driving tumorigenesis. High LIG4 expression was found in prostate tumors harboring the *TMPRSS2:ERG* fusion, a common translocation found in prostate cancers [103]. Furthermore, LIG4 promoter hypermethylation has been shown to contribute to reduced LIG4 expression in colorectal cancer [104]. XLF is overexpressed in HPV(+) HNSCCs and is significantly down-regulated in HNSCC cells expressing high levels of mutant p53 [105].

### 4.3. Single Nucleotide Polymorphisms in Core NHEJ Factors and Carcinogenesis

A host of studies have been directed at identifying genetic single nucleotide polymorphisms (SNPs) in the core NHEJ factors that are associated with carcinogenesis. One of the significant drawbacks to many of these studies is that they had small patient sample sizes and/or were never verified via subsequent studies. Below, we will highlight a number of identified SNPs that likely do correlate with carcinogenesis. The Ku80 polymorphism G-1401T (SNP rs828907) has been correlated with oral, bladder, breast, colorectal, and gastric cancer susceptibility [106,107,108,109,110]. This polymorphism is located in the promoter region of the *Ku80* gene and has been predicted to influence the expression level and/or stability of the Ku80 protein. The fact that this polymorphism has been found to correlate with five different solid tumor types suggests that it is possibly a useful biomarker for cancer detection or patient triage. A number of polymorphisms have been identified in the *XRCC6* promoter that correlate with increased carcinogenesis. The Ku70 C-61G polymorphism (SNP rs2267437) is associated with increased breast cancer incidence, RCC, HCC, prostate, and lung cancer [111,112,113,114,115,116,117,118,119]. The Ku70 promoter T-991C (SNP rs5751129) polymorphism is associated with HCC, gastric, oral, renal, and nasopharyngeal cancer susceptibility, and this novel polymorphism is predicted to lead to differential XRCC6 mRNA and Ku70 protein expression levels [115,120,121,122,123]. The Ku70 A-31G promoter polymorphism (SNP rs132770) is associated with increased renal cancer incidence [123,124]. Surprisingly, the *XRCC6* A46922G polymorphism (SNP rs132793) was found to be protective against breast carcinogenesis but associated with increased incidence of HCC and glioma [111,118,125,126]. Each of these *XRCC6* polymorphisms was verified as affecting overall tumor risk in a systemic review and meta-analysis [127]. Only one *PRKDC* polymorphism, intron 8 G6721T (SNP rs7003908), has been identified to be associated with a significant increase risk in carcinogenesis (glioma, bladder, colorectal, and prostate cancer) [128,129,130,131]. The functional significance of this polymorphism is unknown, but it is predicted that it might regulate splicing and cause mRNA instability or may be a haplotype with other genetic changes in other disease-related genes through a linkage disequilibrium mechanism [129].

Polymorphisms that are associated with carcinogenesis have also been found in LIG4 and XRCC4. A strong association between the LIG4 polymorphism GA+AA (SNP rs10131) and glioma and ovarian cancer incidence has been found [132,133,134]. The XRCC4 intronic polymorphism T1394G (SNP rs2075685) is associated with increased breast, oral, and pancreatic cancer incidence [111,135,136,137,138]. The recessive missense XRCC4 polymorphism at codon 247 (c.739G>T, pAla247Ser) (SNP rs3734091) is associated with development of oral cancer, HCC, and breast cancer, in particular increasing the risk of developing triple-negative breast cancer [139,140,141]. This mutation disrupts XRCC4 nuclear localization and results in reduced DSB repair, increased IR sensitivity, and genomic instability, and has been shown to be associated with an increased risk of metastasis [140,141]. XRCC4 D allele polymorphism (SNP rs28360071) is associated with oral cancer, and this polymorphism was found to be significantly associated with cancer risk in a meta-analysis [142,143]. The XRCC4 polymorphism (SNP rs1805377) (splice site 1) is associated with genetic susceptibility to RCC, glioma, NSCLC, and bladder cancer, and patients with this SNP had significant genomic instability and poor prognosis [117,144,145]. The XRCC4 SNP rs1805377 is also associated with poor survival in patients with metastatic prostate cancer [146]. Carriers of the XRCC4 G-1384T polymorphism (SNP rs6869366) are at a significantly higher risk of developing NSCLC, esophageal, bladder, prostate, gastric, colorectal, and urothelial cancer [147,148,149,150,151,152].

Surprisingly, a number of LIG4 and XRCC4 polymorphisms have also been identified that associate with a decreased risk of cancer. A LIG4 polymorphism has been identified, which results in the amino acid substitutions T9I (SNP rs1805388), that is significantly associated with a reduction in risk in developing multiple myeloma, ALL, and HNSCC, and a meta-analysis found that this polymorphism is associated with a decreased cancer risk among Caucasians [153,154,155,156]. The LIG4 T>C at nt 1977 polymorphism (SNP rs1805386) is significantly associated with a decrease in breast cancer risk and survival [157,158]. Longer survival among patients with non-invasive tumors associated with XRCC4 polymorphism (SNP rs2662238) was found in patients with bladder cancer [159]. A decreased risk of non-astrocytoma brain cancer is associated with XRCC4 polymorphisms SNP rs7721416 and rs2662242 [160].

### 4.4. Differential Expression of Core NHEJ Factors in Cancer Progression and Survival

Differential expression of the NHEJ core machinery has also been found to correlate with cancer progression and overall survival. The majority of the studies have found that increased Ku expression results in increased tumor proliferation/metastasis and decreased survival. For example, up-regulation of Ku70 and Ku80 protein levels was found to correlate with tumor proliferation rate in non-melanoma skin cancer [161]. Increased expression and chromatin bound levels of Ku80 are also implicated as a driver of breast cancer progression [162]. In rectal carcinomas, increased Ku expression correlates with decreased disease-free survival [163]. In endometrial carcinoma, disease-free survival rates were significantly higher in patients with a low percentage of Ku70-positive tumor cells [164]. High expression of Ku80 was associated with significantly lower survival rates in patients with primary melanoma [165]. A univariate analysis found that overall survival and progression-free survival were significantly better in lung adenocarcinoma patients with low vs. high Ku80 expression levels [166]. High expression of Ku80 is correlated with a lower rate of progression-free survival in nasal type NK/T cell lymphoma [167]. High nuclear Ku70/80 expression was correlated with features of poor prognosis including higher histological grade, lymphvascular invasion, negative oestrogen receptor expression, basal-like phenotype, p53, and CHK1 positivity in breast cancers [168]. However, a few studies have found the opposite. High tumor Ku70 mRNA expression was associated with significantly longer local recurrence free survival in patients with HNSCC [169]. In breast cancer tissues, lower expression levels of Ku70 and Ku80 tended to be associated with a higher malignant nuclear grade of cancer cells and higher frequency axillary lymph node metastasis [170]. Advanced high-grade, high-stage urinary bladder carcinomas had lower mRNA Ku70 and Ku80 expression than superficial low-grade, low-stage carcinomas, suggesting that down-regulation of the Ku heterodimer is associated with progression of bladder cancer from a low to a high malignant potential [171]. Finally, down-regulated Ku70 was found to be associated with poor disease-free survival in colorectal cancer patients [172].

Increased expression of DNA-PKcs has been correlated with cancer progression and decreased survival. At the mRNA level, DNA-PKcs expression was significantly higher in NSCLC tumor tissues than in the adjacent normal tissues, with this increase in DNA-PKcs expression being associated with an increased risk of death [98]. Furthermore, DNA-PKcs expression is a prognostic predictor of nasopharyngeal cancer patients with poor survival outcomes and likely plays a role in recurrence and metastasis [173]. Elevated levels of DNA-PKcs in ovarian serious adenocarcinoma tissues are an indication of a more advanced disease and worse prognosis [174]. Increased *PRKDC* mRNA and protein levels were found to be significantly associated with Gleason score, tumor stage, distant metastasis, and predicted poor survival by Kaplan-Meier analysis in prostate cancer [95]. This is supported by a study that reported that positive DNA-PKcs nuclear staining was associated with biochemical recurrence of prostate cancer [175]. DNA-PKcs has also been classified as a candidate driver in hepatocarcinogensis, with amplification of the *PRKDC* locus, increased mRNA expression, and increased autophosphorylation at serine 2056 correlating with poor survival [100]. In the case of serous cystadenocarcinomas and ovarian cancer, high DNA-PKcs mRNA and protein levels correlated with poor survival [176]. Preclinical findings have also identified DNA-PKcs as a potential driver of pro-metastatic signaling via transcriptional regulation in castration-resistant prostate cancer [177]. Upregulation of DNA-PKcs predicts tumor metastasis, recurrence, and poor survival, and it is highly active in metastatic tumors, independent of DNA damage indicators [177]. In the case of melanoma, DNA-PKcs stimulates angiogenesis, migration, and invasion, providing strong evidence that it may provide a pro-metastatic effect on the tumor microenvironment [178]. Increased DNA-PKcs and epidermal growth factor receptor (EGFR) activities are positively correlated, and may contribute to metastatic phenotype and therapeutic resistance in human cancer cells [179]. Finally, DNA-PKcs has been shown to regulate Snail1, an inducer of epithelial to mesenchymal transition [180]. Collectively, there is significant evidence that DNA-PKcs plays a role in aggressive primary tumors and promotes metastasis.

There is also evidence that decreased DNA-PKcs expression can also drive tumor progression. Breast cancer patients with lower DNA-PKcs levels tended to have a higher rate of distant metastases and poorer prognosis [181]. The DNA-PKcs activity in the PBLs of patients with advanced cancer was determined to be significantly lower than in patients at an earlier cancer stage, and those patients had lower rates of disease free and distant metastasis free survival [182]. In gastric cancers, decreased DNA-PKcs expression is associated with tumor progression, lymphatic involvement, advanced pTNM stage, and poor survival in gastric cancers [183,184]. Decreased expression of DNA-PKcs may drive cancer outcomes due to abrogation of NHEJ-mediated DSB repair and a subsequent increase in the aberrant Alt-EJ pathway, resulting in increased genomic instability.

Only a limited number of studies have found a correlation between expression of LIG4, XRCC4, or XLF and cancer progression and patient outcomes, but a couple of studies have found that increased expression of these factors results in increased aggressiveness in tumors. High LIG4 expression was found to be tightly linked to advanced Gleason score, positive nodal involvement, and aggressiveness in prostate cancer [103]. High mRNA expression of XRCC4 was found to be prognostic of poor outcome for patients with ovarian cancer [185].

### 4.5. Differential Expression of the Core NHEJ Factors and Its Influence on Therapy Responsiveness

A significant amount of data has been generated that shows that tumors with differential expression of the core NHEJ machinery have differential therapy responsiveness. Decreased expression of the NHEJ core factors has been found to result in increased responsiveness to cancer treatments. Ovarian cancer tissues that showed decreased Ku70 expression were hypersensitive to DNA damage and showed increased cell killing with low doses of IR with the addition of two PARP inhibitors (PJ34 and olaparib) [186]. In cervical carcinomas, a correlation between measurements of tumor radiosensitivity and Ku70 expression was found, as all tumors with a lower number of Ku70- or Ku80-positive cells were radiosensitive [187]. Patients with squamous cell carcinoma of the hypopharynx (stages I–III) that had lower expression of Ku70 or XRCC4 tended to have better locoregional control that correlated with increased radiosensitivity [188]. A unique case of Ku70 regulating clinical outcomes is in castration-resistant prostate cancer. It was found that after castration, Ku70 expression and subsequently NHEJ is significantly decreased, which explains the improved response of patients with prostate cancer to radiotherapy after chemical castration [189,190]. In a study by Harima et al., Ku80-negative cervical cancer tumors showed significantly better response rates than Ku80-positive tumors with better overall survival in the Ku80-negative patients [191]. Furthermore, patients with low DNA-PKcs expression had a greater benefit from radiotherapy for NSCLC than patients with high levels [192].

It has also been found that increased expression of the NHEJ machinery results in resistance to conventional therapies. Increased Ku70 and Ku80 expression was found to result in tumor radioresistance in rectal carcinomas [163]. Increased Ku80 expression was found to correlate with increased resistance to cisplatin chemotherapy in lung adenocarcinoma patients [166]. Ku80 is overexpressed in half of all HNSCC tumors analyzed in a study Moeller, B.J., et al., and it was found to be an independent predictor for both locoregional failure and mortality following radiotherapy [193]. Increased DNA-PKcs expression predicted poor outcome in nasopharyngeal carcinoma patients undergoing intensity modulated radiation therapy (IMRT), independent of classical markers [181]. DNA-PKcs also promotes radiation resistance in cervical and breast cancers as well as correlating with radioresistance in lung cancer lines [194,195,196]. DNA-PKcs activity also correlated with resistance to cisplatin in glioma [197] and chemoresistance in B-cell chronic lymphocytic leukemia (CLL) [198]. Following irradiation of oral squamous cell carcinomas (OSCC), the expression of DNA-PK and associated proteins correlated with tumor radioresistance, suggesting that up-regulation of DNA-PKcs following irradiation conveys radioresistance [199]. Increased expression of DNA-PKcs conveyed resistance to brachytherapy in patients with localized prostate cancer [200]. Finally, it was demonstrated that DNA-PKcs expression correlated with greater therapeutic sensitivity in esophageal cancer [201]. Wnt/β-catenin signaling enhances LIG4 expression, and upregulation of LIG4 plays a key role in radioresistance in tissue stem cells and colorectal cancer cells [202]. Oral cancer stem cells display resistance to IR, and this correlates with elevated levels of XLF [203]. Collectively, the data suggest that the expression level of the core NHEJ factors impacts therapy responsiveness, with decreased expression resulting in radiosensitivity due to the inability of the cells to properly deal with DSBs, and increased expression resulting in radioresistance.

## 5. Manipulating NHEJ as an Anti-Cancer Therapy

Conventional, non-surgical cancer treatment is founded on the utilization of clastogenic agents, which include ionizing radiation (IR) and chemotherapy, to induce DNA damage. This DNA damage, particularly DNA DSBs, results in lesions that are more cytotoxic to quickly dividing tumor cells than to the more slowly or non-dividing surrounding normal tissues. This difference in response to DNA damage between cancer cells and normal tissues thus provides a therapeutic advantage to IR and chemotherapy in targeting cancerous lesions. Despite the relative efficacy of these agents in treating a wide variety of cancers, there are still unresolved issues regarding the therapeutic resistance of both primary and recurrent tumors. Furthermore, the toxicity of normal tissues exposed to IR or chemotherapy remains a concern. Therefore, much time and interest have been devoted toward the development of concomitant therapies that are capable of sensitizing tumor cells to conventional therapeutics or that protect normal tissues. The sensitization of tumor cells to conventional non-surgical therapy enables smaller quantities of the cytotoxic agent to be delivered to achieve the same cure rate, thus reducing normal tissue damage. Conversely, protecting normal tissues allows for greater amounts of the cytotoxic agent to be delivered to the tumor, increasing the probability of tumor cure without additional normal tissue complications. The integral role of DNA DSB repair pathways in the survival of cells exposed to genotoxic agents represents a promising target to sensitizing tumors, further improving the therapeutic ratio and enhancing cancer therapy. The sensitization of tumor cells, in particular to IR, through the inhibition of NHEJ, is a promising approach. Modern radiotherapy techniques, including stereotactic ablative/body radiation therapy (SABR, SBRT), proton therapy, and heavy ion radiotherapy, deliver a radiation dose to the tumor while minimizing the dose delivered to the surrounding normal tissue structures. Thus, minimizing the risk that inhibiting NHEJ will lead to higher rates of normal tissue injury. Furthermore, the potential exists for dose de-escalation when using NHEJ inhibitors that sensitize the tumor to radiation, leading to higher cure rates at lower doses. In this section, we will present the rationale for and give an overview of the various strategies currently used to target and inhibit the core NHEJ factors for the purposes of concomitant cancer therapy and small molecules that target the core NHEN factors are listed in Table 1.

### 5.1. DNA-PKcs

A large body of evidence has confirmed the vital role that DNA-PKcs plays in the survival of cells exposed to DSB inducers. Lack of expression or siRNA knockdown of DNA-PKcs leads to enhanced sensitization of cells to DSB inducing agents, such as IR, topoisomerase I and II inhibitors, and nitrogen mustard gas [204,205,206,207,208,209,210,211,212,213,214]. As previously noted, DNA-PKcs is upregulated in radioresistant cancer cell lines, and this upregulated DNA-PKcs activity may promote therapeutic resistance [196,215,216,217,218,219,220,221]. Taken together, these studies provide a strong rationale that DNA-PKcs is a potential therapeutic target.

Several approaches have been undertaken to target DNA-PKcs by decreasing its protein expression, inhibiting its recruitment to the Ku70/80:DNA complex, and most notably, chemically inhibiting its kinase activity [204]. Decreased expression with anti-sense oligonucleotides against DNA-PKcs has been demonstrated [209,212,222], however these methods remain outside the scope of clinical practice, and thus do not represent a promising therapeutic approach. Blocking the binding of DNA-PKcs to the Ku70/80:DNA complex is another approach for inhibiting its role in NHEJ. Ku80 recruits DNA-PKcs to DSBs via its C-terminal domain, and stable expression of this C-terminal domain in cells prevents DNA-PKcs recruitment to DSBs and acts in a dominant-negative fashion [222]. Furthermore, peptides targeting the Ku-DNA-PKcs binding interface sensitize cells to chemotherapy and IR, indicating their potential clinical utility [223]. While this approach is technically feasible, expressing or delivering synthetic peptides to a tumor does not represent an immediately clinically applicable approach.

The most promising approach to inhibiting DNA-PKcs activity remains targeting the ATP binding pocket in its PI3K-like kinase domain via small molecules. The first reported inhibitor of DNA-PKcs kinase activity, caffeine, sensitizes cells to IR, however its low affinity relative to other PIKK kinase family members precluded its application in further experimentation [224]. Another ATP non-competitive inhibitor, wortmannin, was found to inhibit not only PI3K, but also the PIKK family members DNA-PKcs, ATM, and ATR [225]. However, the half minimal inhibitory concentration (IC_50_) for wortmannin for DNA-PKcs is two orders of magnitude higher than for PI3K, illustrating that this inhibitor is not specific for DNA-PKcs. This is further supported by studies that show that wortmannin concentration needs to be extremely high to result in in vivo tumor radiosensitization [226,227,228]. Additionally, wortmannin’s poor water solubility makes it an unattractive candidate for clinical use.

Additional inhibitors of DNA-PK include those molecules derived from the LY294002 family, a synthetic derivative of quercertin that inhibits as a competitive ATP binding inhibitor on DNA-PKcs [229]. However, at DNA-PK inhibiting concentrations, LY294002 also inhibits CKII, a kinase involved in multiple signaling pathways. The crystal structure of porcine PI3Kγ has been solved in complex with wortmannin and LY294002, providing the information needed to synthesize more specific inhibitors [230]. From this approach, LY294002 was utilized as a lead compound to generate NU7026 with a concentration of 10 µM necessary to sensitize cells to IR or chemotherapy in vitro [231,232], which is still too high to be clinically relevant. Expanding on this approach, the more selective inhibitor NU7441 was developed and is currently under evaluation [233]. Furthermore, the compound SU11752 was found to inhibit DNA-PKcs through competition with ATP and has an IC_50_ of 0.13 µM; at 12 µM concentrations it inhibits DSB repair, and at concentrations exceeding 50 µM, it displays a 5-fold radiosensitization of cancer cells in vitro [234]. However, it also inhibits p110γ, confounding the mechanism of radiosensitization. Other DNA-PKcs inhibitors studied in vitro or in vivo include: vanillin [235,236], OK-1035 [237,238], and phosphatase inhibitors [239]. Vanillin and its derivatives sensitized cells to hydrogen peroxide and mitomycin-C, and they suppressed X-ray induced chromosome aberrations (which result from rejoining via NHEJ) [235,236]. OK-1035 is an effective DSB repair inhibitor but displayed poor inhibition with an IC_50_ value of 100 µM [237,238]. Finally, phosphatase inhibitors were utilized to block DNA-PK activity, but the mechanism is largely unknown [239]. The common problem encountered with all these inhibitors is that the high efficacy in vitro is not seen in vivo, likely due to low drug uptake.

Two predominant strategies to utilize DNA-PKcs inhibition as a clinical cancer therapy are under investigation: one is utilizing these inhibitors as radiosensitizers, the other being the potential for synthetic lethality. Radiation therapy targets radiation dose to the tumor while sparing normal tissues; therefore, systemic administration of DNA-PKcs inhibitors can prevent repair of tumor DNA damaged in the irradiated field, while being largely irrelevant in normal tissues which may have temporary DNA-PKcs inhibition but do not experience DNA DSBs since they are not in the radiation field. This is of importance because cell lines from two radiation therapy patients who later went on to develop radiation necrosis as a treatment induced late effect were found to lack DNA-PKcs expression [240]. Following this strategy, two Phase 1a/1b clinical trials utilizing the DNA-PKcs kinase inhibitor MSC2490484A are currently recruiting patients to utilize this compound in combination with either radiation therapy or chemoradiation therapy (IR + cisplatin) for locally advanced solid tumors of the head and neck or thoracic region (NCT02516813). Furthermore, clinical trial NCT02316197 seeks to utilize MSC2490484 in locally advanced, solid tumors or CLL that contain altered DNA repair mechanisms. Both of these studies are first-in-man safety studies to determine the tolerability and pharmacokinetic profile of these agents, with secondary endpoints being efficacy and tumor cure. A third Phase 1a/1b clinical trial is ongoing, but no longer recruiting, utilizing a compound named CC-115, a dual DNA-PKcs and mTOR kinase inhibitor, to assess the safety of this compound in patients with advanced solid tumors or CLL (NCT01353625). An additional clinical trial utilizing the dual DNA-PKcs/mTOR inhibitor CC-115 in combination with enzalutamide for the treatment of castration-resistant prostate cancer (NCT02833883) is currently ongoing in patients screened for a specific set of biomarkers. Applying DNA-PKcs inhibitors in generating synthetic lethality in ATM-defective tumors, HR deficient tumors, and MSH3 deficient tumors are recent areas of interest in this realm [241,242]. Furthermore, the clinical trial NCT02977780 is utilizing CC-115 in brain tumor patients screened for specific biomarkers to identify specific molecular pathways that may indicate the potential for synthetic lethality when DNA-PKcs inhibitors are utilized. Collectively, while still in its infancy, the applied use of DNA-PKcs kinase inhibitors in the clinic remains a promising candidate for adjuvant cancer therapy.

### 5.2. Ku70/80

The Ku70/80 heterodimer is the central regulator of NHEJ. The Ku heterodimer binds to the DSB ends and recruits the NHEJ machinery to process and ligate the DSB. Ku’s central role in NHEJ is supported by the evidence that either Ku70 or Ku80 deficiency leads to profound sensitization to both IR and radiomimetic chemotherapeutics [40,243,244,245]. However, because Ku’s activity does not rely on kinetic activity, and the central DNA binding canal of the Ku70/80 heterodimer is smooth, targeting Ku with small molecules is challenging [245,246]. Recently, Weterings et al. described the identification of a series of small molecules that targets the Ku DNA binding pocket [247]. One of these compounds (Compound L) blocks Ku70/80:DNA binding and prevents the activation of DNA-PKcs. Furthermore, the compound sensitizes two human cancer cell lines to IR. This initial study illustrates that blocking the binding of Ku70/80 to the site of DSBs via small molecule inhibitors represents a promising approach, but further in vivo and in vitro testing is necessary prior to moving this compound into clinical trials.

### 5.3. LIG4

Targeting LIG4 and thus inhibiting the terminal ligation step of NHEJ would require blocking the recruitment of LIG4 to DNA ends, disrupting the LIG4/XRCC4 interaction, or inhibiting its ligase activity, which has practical hurdles due to the similarities between LIG4 and the other human DNA ligases, LIG1 and LIG3 [10]. Targeting LIG4 is of interest because inhibitors to LIG4 will result in increased radiosensitivity and because over-expression of LIG4 has been shown to result in radioresistance. A small molecule targeting LIG4’s ability to bind DNA ends known as SCR7 was identified by Srivastava et al. [248]. This novel inhibitor suppressed NHEJ and significantly sensitized xenograft animal tumors to IR and etoposide. This study identified the first molecule capable of suppressing NHEJ by blocking the binding of LIG4 to DNA ends. However, it was recently published that SCR7 is neither a selective nor potent inhibitor of human LIG4, which brings this inhibitor into question [249]. Utilizing the crystal structure of the Lig4/XRCC4 complex, a recent study identified a compound that was able to prevent Lig4 binding to XRCC4 in vitro, suggesting that blocking the ability of Lig4 to interact with XRCC4 is a potential viable drug target [250]. Finally, the compound L189 was identified that competitively inhibits DNA Ligases I, III, and IV [251]. In cell culture assays, L189 was found to be cytotoxic, but using subtoxic concentrations of L189 significantly sensitized cancer cells to IR, suggesting that this compound could be used for the development of anticancer agents [251].

## 6. Perspective on the NHEJ Conundrum and Concluding Remarks

It has been clear for decades that NHEJ promotes genomic stability via its ability to mediate DNA DSB repair. As a protector of the genome, loss of the NHEJ machinery should drive chromosomal aberrations, resulting in carcinogenesis. However, complete loss of the core NHEJ machinery is not found (or is rarely found) in humans. We postulate that NHEJ plays a much more significant role in genome maintenance in humans than in lower organisms, that it is responsible for the repair of the vast majority of two-ended DSBs in all cell cycle phases. Furthermore, we speculate that complete loss of the core NHEJ factors would result in a high DSB burden that would ultimately drive increased apoptosis. Ku70/80 and DNA-PKcs are also important for telomere maintenance, which likely also plays a role in their overall importance in human cells. However, we postulate that mutations in the core NHEJ machinery result in abrogation or attenuation of the NHEJ pathway, resulting in genomic instability and carcinogenesis. Cancer genome studies have identified a significant number of mutations in the core NHEJ machinery factors, with most listed in the Sanger Institute Catalogue of Somatic Mutations in Cancer (COSMIC) database (http://www.sanger.ac.uk/) and the NIH/NCI TCGA database (http://www.cbioportal.org) [258,259]. Individual groups sequencing specific cancers have also identified mutations in the core NHEJ factors, such as the study that performed whole-exome sequencing on 109 pancreatic cancers, which found that *XRCC4* is mutated or deleted in 4% of the pancreatic tumors [260]. A study examining the mutational landscape across 12 cancer types found a significant correlation between *PRKDC* mutations and overall high mutation frequency in bladder urothelial carcinoma, colorectal carcinoma, lung adenocarcinoma, and uterine corpus endometrial carcinoma [261]. Furthermore, a report analyzing the genomic landscape of DNA repair genes in cancers by examining a comprehensive list of DNA repair genes and mutations, copy number variations (CNV), and expression frequencies found in the COSMIC database, with mutation co-occurrence, clinical outcomes, and mutation burden analyzed in the TCGA database, was published [262]. The authors found that *PRKDC* was the sixth most frequently mutated repair gene in all common cancers with *PRKDC* mutated 2.1% of the time in all cancers. In regards to individual cancer types, it is mutated 4.0% in lung, 1.5% in breast, 1.9% in liver, 12.1% in large intestine, 5.9% in colorectal, and 5.5% in skin cancer. The authors found that somatic mutation burden in colorectal tumors correlated with *PRKDC* mutation and that this resulted in an increased overall mutation burden. Finally, the authors found that *PRKDC* and *LIG4* both had significant CNV gains across all tumor types. Lastly, high mutation burden was associated with a nonsense mutation in *PRKDC* in non-small cell lung carcinoma, and tumors with a high mutation burden were associated with increased sensitivity to the immune checkpoint inhibitors [263]. Identifying and differentiating between driver and passenger mutations in the core NHEJ factors is a difficult endeavor, albeit one that this is a worthwhile task.

NHEJ has traditionally been described as error-prone, because it does not use a homologous template to drive repair. If the DSB ends are not compatible, NHEJ-mediated repair can result in small deletions, insertions, or indels, but NHEJ is likely much more precise than previously believed due to the flexibility of the NHEJ factors. We postulate that NHEJ drives chromosomal aberration formation, in particular those that are believed to result in carcinogenesis, such as translocations, when the HR and FA pathways are deficient [264,265]. The HR and FA pathways typically block NHEJ from mediating the repair of one-ended DSBs, because NHEJ-directed repair of these types of breaks results in chromosomal aberrations. However, when one-ended DSBs, such as replication associated DSBs, are generated in an HR- or FA-deficient cell, we believe the cell utilizes NHEJ to correct the DSB, because the cell would rather have a chromosomal aberration generated than have an unrepaired DSB, because even one unrepaired DSB can result in cell death [2]. In this regard, NHEJ becomes the DSB repair pathway of last resort. Therefore, NHEJ may drive genomic instability in human cells, but it does so the majority of the time in order to rescue the cell. Furthermore, we believe that HR- and FA-deficient cancer cells become “addicted” to NHEJ. We postulate that these cells overexpress the core NHEJ factors to ensure that there is adequate NHEJ-mediated repair to compensate for the loss of the HR or FA pathways to assist the cancer cell in dealing with the large number of DSBs generated during rapid cell proliferation, which is found in tumors. We also speculate that increased DNA-PKcs expression compensates for ATM- and ATR-deficiency in a number of malignancies. Tumor cells that are overly dependent on the NHEJ pathway, in particular those that overexpress the core NHEJ factors, could potentially be targeted with therapies directed at the NHEJ pathway [202,241,242,264,266]. Taken together, we believe that the NHEJ conundrum should be viewed in the context of the cell, with normal cells using NHEJ to stabilize the genome, but precancerous and cancer cells utilizing NHEJ to drive genomic instability and carcinogenesis.

In conclusion, in this review, we presented a comprehensive view of NHEJ in the context of cancer by discussing alterations in the expression pattern or point mutations in the core NHEJ factors and how they potentially drive genomic instability, carcinogenesis, tumor grade and aggressiveness, response to therapy, and prognosis. We also presented evidence that selective, concomitant targeting of these misregulated NHEJ factors provides a potential therapeutic target for sensitizing cancers resistant to conventional chemo and radiation therapy, and therefore provides greater therapeutic outcomes. Finally, we presented a perspective to reconcile NHEJ’s seemingly contradictory role in maintaining genomic instability, however when misregulated or in the context of additional DNA repair defects, promoting and driving carcinogenesis.

## Figures and Tables

**Table 1:** List of compounds targeting the core NHEJ factors.

Molecular Target	Compound Name	IC_50_	References
DNA-PKcs	Caffeine	ATM: 1.2 mM	[224]
		ATR: 1.1 mM	
		DNA-PKcs: 10 mM/L	
	Wortmannin	ATM: 150 nM	[252]
		ATR: 1.8 μM/L	
		DNA-PKcs: 16 nM/L	
	LY294002	PI3K: 1.4 μM	[229]
	KU55933	ATM: 13 nM	[253]
		ATR, DNA-PKcs: 16 nM/L	
	CP466722		[254]
	NU7026	DNA-PKcs: 230 nM	[231]
		ATM, ATR: 13 μM	
	NU7441	DNA-PKcs: 230 nM	[214,233,255]
		ATM, ATR: ≥100 μM	
	KU0060648	DNA-PKcs: 0.019–0.17 μM	[256]
	MSC2490484A		NCT02316197
			NCT02516813
	VX-984		NCT0264427
	CC-115		NCT01353625
			[257]
Ku70/80	STL127705		[247]
DNA Ligase IV	SCR-7		[248,249]
	Compound #3101		[250]
